# Inflammatory dietary scores and their association with clinical outcomes in coronary heart disease

**DOI:** 10.3389/fnut.2026.1788305

**Published:** 2026-04-13

**Authors:** Ruxia Zhang, Sancong Pan, Huahua Cui, Ganggang Si, Jianjun Li, Xiangbing Li, Chunguang Qiu

**Affiliations:** 1 Department of Cardiology, The First Affiliated Hospital of Zhengzhou University, Zhengzhou, China; 2 Department of Cardiology, Jincheng People’s Hospital, Jincheng, Shanxi, China

**Keywords:** cardiovascular mortality, coronary heart disease, Dietary Inflammatory Index, dietary pattern, inflammation, major adverse cardiovascular events (MACE)

## Abstract

**Background:**

Chronic inflammation is a focal process in the pathophysiology and pathogenesis of coronary heart disease (CHD). Dietary Inflammatory Index (DII) has been demonstrated as a potential useful marker to identify individuals at higher risk of adverse outcomes in CHD patients.

**Aim:**

This paper examined how dietary inflammatory potential is related to negative clinical outcomes in the population with angiographically confirmed CHD.

**Methods:**

This is a retrospective cohort study that involved 500 adults with CHD, who were followed up a median of 38 months. The baseline dietary intake was measured, and the DII scores were computed and placed in quartiles. Major adverse cardiovascular events (MACE), death by any cause, and cardiovascular-related re-hospitalization were registered. The associations between DII and clinical outcomes were estimated with the help of cox proportional hazards and logistic regression models that took into consideration demographic, clinical, and lifestyle confounders. The correlation of DII and circulating inflammatory biomarkers were also studied.

**Results:**

A positive association was found between higher DII scores and the negative cardio-metabolic phenotype and high systemic inflammation. The incidence of MACE over time was more and more increased with DII quartiles (11.2% in Q1 and 29.6% in Q4; *p*-trend <0.001). Once fully adjusted, the participants in the top quartile of DII had a much greater probability of MACE (HR 1.82, 95% CI 1.27–2.61) and of all-cause mortality (HR 1.68, 95% CI 1.05–2.69) than the participants in the lowest quartile. Every one unit rise in DII corresponded to a 21 percent increase in the risks of MACE. Greater DII scores were also associated with higher cardiac-related hospital readmission rates and high levels of inflammatory biomarkers, such as hs-CRP.

**Conclusion:**

Pro-inflammatory dietary pattern (high DII scores) are associated with adverse cardiovascular outcomes and mortality in this cohort. Such results indicate that dietary inflammation is an associated factor in CHD outcomes, and further research is warranted to examine whether modifying dietary inflammatory potential could influence clinical outcomes.

## 1 Introduction

Coronary heart disease (CHD) remains a significant global issue in terms of population health as it causes significant mortality, long-term disability, and healthcare cost. Recent epidemiological findings suggest that CHD is caused by the interplay between conventional risk factors, including hypertension, diabetes mellitus, dyslipidemia, and smoking, with behavioral and environmental factors, especially diet (1). In addition to lipid deposition, CHD is currently well known as a chronic inflammatory disease where endothelial dysfunction, immune cell infiltration, and atherosclerotic plaque gradual accumulation takes place.

The eating habits are essential in the regulation of the systemic inflammation. In order to measure the cumulative capacity of dietary habit to drive inflammatory response, the Dietary Inflammatory Index (DII) was created as a literature-based instrument that measures the pro- or anti-inflammatory content in dietary intake. An increase in the scores of DII is a sign of a high intake of refined carbohydrates, saturated fats, and processed foods, and the reverse is also true. First population-based indicators revealed a strong correlation between a high level of DII scores and the existence of CHD, which indicates that dietary inflammation is an important factor in coronary pathology (2). These results have been strengthened by large-scale observational studies performed on different populations. Research studies on tens of thousands of adults have revealed that patients with increased scores on DII demonstrate much higher prevalence of CHD after controlling traditional cardiovascular risk factors (3). These findings confirm the hypothesis that inflammatory nutritional habits could have an independent effect on CHD, regardless of the established metabolic and lifestyle factors.

Mechanistic and biomarker-based studies are increasingly providing the biological plausibility of these associations. The dietary inflammation has been attributed to an increase in circulating inflammatory mediators, oxidative stress, and immune responses. Complex methods to combine dietary intake with inflammatory biomarkers have shown that nutritional exposures have systemic inflammatory outcomes, which can put people at risk of developing chronic disease by acting on common inflammatory pathways (4). These processes provoked by inflammation are key to endothelial damage, the development of the plaque, and the subsequent destabilization of the plaque. Notably, the effect of dietary inflammatory potential is not confined to individual coronary disease but is strongly interconnected with the total cardio-metabolic malfunction. It has been identified that elevated DII scores are linked to the cardiovascular-kidney-metabolic syndrome a set of interrelated disorders, such as insulin resistance, hypertension, renal impairment, and dyslipidemia, which together increase cardiovascular risk (5). The same results of independent cohorts also prove that pro-inflammatory diets can also contribute to systemic metabolic dysregulation that paces up the process of atherosclerotic disease (6).

There are sex- and ethnicity-specific studies, which point out further aspects of dietary inflammation. The longitudinal data of large groups of females show that the greater the inflammatory potential of a diet, the higher the risk of cardiovascular disease, which proves the importance of diet-related inflammation in demographic sub-groups and the necessity to develop dietary intervention that suits a population (7). Although the literature on the topic is growing, there are still some critical gaps. According to bibliometric analyses, the number of investigations in the field of dietary behavior and cardiovascular research increases very quickly, but there are only relatively fewer studies examining inflammatory dietary indices in clinical outcomes among patients who have developed CHD (8). In addition, systematic reviews have shown that, although dietary inflammatory indices are always linked with inflammatory biomarkers, minimal evidence exists which links these indices concomitantly with biomarkers and longitudinal cardiovascular outcomes (9). Meta-analytic data also provide evidence of large heterogeneity between studies, which implies that clinically oriented studies should be conducted that measure hard cardiovascular outcomes and not only disease prevalence (10).

The current retrospective cohort study sought to examine the relationship between dietary inflammatory potential, measured by Dietary Inflammatory Index, and clinical outcomes in subjects with angiographically verified coronary heart disease. Although previous research has identified DII and CHD occurrence, limited research has been conducted to establish the relationship between DII, inflammatory biomarkers, and extended clinical outcomes (MACE, all-cause mortality, cardiac readmissions) in a cohort of patients with angiographically verified CHD. Also, a lot of the studies failed to correct all possible confounding factors such as socioeconomic status, lifestyle choices, and medication compliance. The study thus aims at investigating these relations in a well-characterized cohort.

## 2 Methodology

### 2.1 Study design

The paper was formulated as a retrospective cohort study that attempts to determine the relationship between inflammatory dietary scores and clinical outcomes in coronary heart disease (CHD) patients. The research was undertaken in a tertiary-care cardiology setting based on clinical data gathered on a regular basis and stored dietary examinations. The patients with the diagnosis of CHD who received the management in the time frame between January 2018 and December 2024 had their electronic medical records reviewed. The baseline data such as diet intake, demographic data, clinical history, laboratory parameters, and medication use were gathered at the time of the first nutritional assessment or hospitalization and preceded the incidence of clinical outcomes. The participants were tracked longitudinally with the help of medical records that were available to determine adverse cardiovascular events and mortality. This research was approved by the Ethics Committee of Jincheng People’s Hospital, Ethical Number: JCPH. No. 20250901002.

### 2.2 Study population

The sample used in the study included 500 adult patients [with a background of coronary heart disease (CHD)] that were selected by retrospectively reviewing electronic medical records in a tertiary-care cardiology center. The participants were eligible and aged 18 years or above and with a diagnosis of CHD confirmed by coronary angiography that showed at least 50 percent stenosis of one or more of the major coronary arteries. Patients who had a baseline dietary assessment within a period of clinical outcome occurrence and full clinical, laboratory, and follow-up data were only included. Patients with chronic inflammatory/ autoimmune conditions, active malignancy, acute inflammatory or infectious diseases at baseline, severe hepatic or renal dysfunction, implausible self-reported energy intake, defined as <500 kcal/day or >5,000 kcal/day, consistent with established nutritional epidemiology criteria (11), or absence of key covariates were excluded. Median follow-up of 38 months of all participants was done to provide sufficient evaluation of clinical outcomes.

### 2.3 Study flow diagram

**Figure 1:**
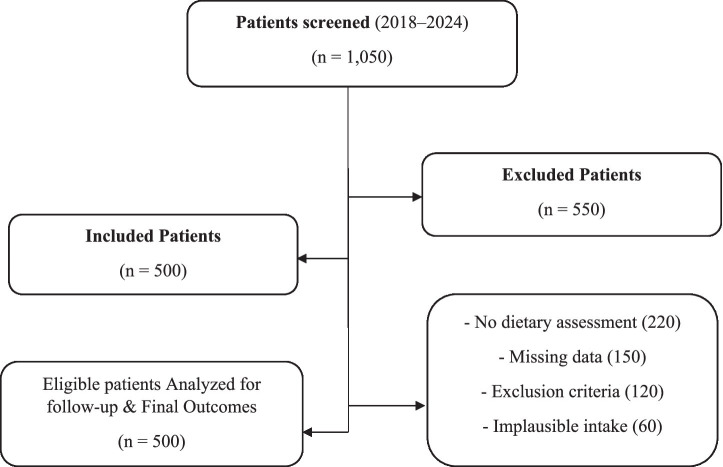
Flow diagram of participant selection and exclusions.

A flow chart of the selection of participants is provided in Figure 1. Electronic medical records were screened on 1,050 patients who had a diagnosis of coronary heart disease between January 2018 and December 2024. Out of them, 550 patients were eliminated due to the following reasons:

- Lack of baseline dietary evaluation (*n* = 220).
- Missing covariate or follow-up data (*n* = 150).
- Existence of exclusion criteria, chronic inflammatory disease, malignancy, or severe organ dysfunction (*n* = 120).
- Unreasonable self-reported energy intake (<500 or >5,000 kcal/day) (*n* = 60).

The other 500 patients who had satisfied the eligibility criteria were included in the final analysis. Every participant who was incorporated had full baseline information and was longitudinally tracked regarding clinical outcomes.

### 2.4 Dietary assessment

Food consumption was determined based on a validated semi-quantitative Food Frequency Questionnaire (FFQ) that was given to the respondents by trained interviewers as a part of standard clinical assessment. The FFQ used in this study has been validated in adult Chinese populations, demonstrating acceptable reproducibility and correlation with 3-day dietary records (12). FFQ was used to record daily successive dietary practice in the last 12 months and has been validated to be used among adult populations. Standard food composition tables were used to calculate nutrient intakes and residual method used to adjust the total energy intake.

### 2.5 Dietary Inflammatory Index (DII)

The Dietary Inflammatory Index (DII) was estimated using the standardized approach established by Shivappa et al. (12). Each of the individual dietary components was standardized into a world reference database, and weighted by its score in terms of inflammatory effect. Out of the 45 food parameters used to calculate the DII, 38 components were available in our dataset, which is consistent with previous studies using partial DII calculation (13). A more pro-inflammatory diet is represented by high DII values and an anti-inflammatory diet pattern is represented by low (negative) values. The participants were identified into quartiles (Q1–Q4) of DII distribution with Q1 as the most anti-inflammatory and Q4 the most pro-inflammatory. Participants reporting implausible total energy intake (<500 kcal/day or >5,000 kcal/day) were excluded, consistent with established nutritional epidemiology criteria (11).

### 2.6 Covariates

The medical records were used to extract baseline covariates comprising of demographic characteristics, lifestyle factors, clinical comorbidities, medication use, and laboratory measurements. The demographic variables were age, sex and race/ethnicity. Race/ethnicity was marked off as Han Chinese. Since the population is fairly homogeneous, this variable was mostly employed descriptively and was not a variable in multivariate models. Lifestyle aspects were smoking status (current smoker vs. non-smoker). BMI derived was used as the basis of anthropometric measurement using the measured weight in kilograms/square and height in meters (kg/m^2^).

Baseline clinical comorbidities were hypertension, diabetes mellitus and dyslipidemia. Standard diagnostic criteria were used in defining clinical comorbidities. Hypertension was considered to be abnormal systolic blood pressure ≥140 mmHg, abnormal diastolic blood pressure ≥90 mmHg or the use of antihypertensive drugs. Diabetes mellitus was the condition that was characterized by fasting plasma glucose ≥126 mg/dL, glycated hemoglobin (HbA1c) ≥6.5% or taking glucose-lowering medication. Dyslipidemia was considered as having total cholesterol ≥200 mg/dL, low-density lipoprotein (LDL-C) ≥130 mg/dL or lipid-lowering therapy. The use of medication such as statins, antiplatelet agents, and antihypertensive agents was recorded to be a possible confounding factor on pharmacological therapy, because these may have an impact on cardiovascular outcome and inflammatory state. The measurement in the laboratory parameters was high-sensitivity C-reactive protein (hs-CRP) to measure systemic inflammation, lipid profile elements (total cholesterol and LDL-C), fasting plasma glucose, and HbA1c. The variables were chosen on the basis of their proven relations with dietary habits, inflammation, and risk of cardiovascular.

Socioeconomic variables, including education level and income status, were collected where available. However, due to incomplete data, these variables were not included in the primary multivariable models to avoid bias. Their potential confounding effect was considered when interpreting results. Although multiple confounders were adjusted for, residual confounding from unmeasured factors such as physical activity, overall diet quality, socioeconomic status, and medication adherence cannot be excluded.

### 2.7 Clinical outcomes

Major adverse cardiovascular events (MACE) or a composite end-point that included non-fatal myocardial infarction, stroke, coronary revascularization procedures, including percutaneous coronary intervention (PCI) and coronary artery bypass grafting (CABG), and cardiovascular deaths were the major clinical outcomes. The follow-up period resulted in secondary outcomes such as all-cause mortality and hospital readmission due to cardiac reasons. Systematic review of the hospital records assisted in extracting clinical outcomes that were validated by standardized diagnostic criteria. Time to first event was estimated as the time interval between date of the baseline dietary assessment to date of outcome occurrence or end of follow-up whichever came earlier.

### 2.8 Sample size considerations

According to past research, which reported hazard ratios of 1.5 to 2.0 between the high and low groups of DII among cardiovascular populations, a sample size of 500 had sufficient power (>80) to statistically identify clinically significant relationships at a two-sided a level of 0.05.

### 2.9 Statistical analysis

The statistical data were processed with the help of the SPSS. The most frequently used techniques to summarize continuous variables were means and standard deviations or medians and interquartile ranges, based on the data distribution, and frequencies and percentages used to provide summaries of categorical variables. Comparisons of baseline characteristics were done between the quartiles of Dietary Inflammatory Index in one-way analysis of variance or Kruskal-Wallis tests, continuous variables, and chi-square tests, categorical variables. Time-to-event results were analyzed as dietary inflammatory score associations with time-to-event using Cox proportional hazards regression models expressed as hazard ratios and 95% confidence interval. Serial models were developed with gradual influence on the variables of demographics, lifestyle, clinical comorbidity, medication intake, and overall energy intake. Linear trend tests were done by modeling DII quartiles as ordinal variables. The sensitivity analyses eliminated the events that happened during the first 6 months of follow up to reduce the possibility of reverse causation. A two sided *p*-value of below 0.05 was regarded as statistically significant. Socioeconomic variables were not included in the primary models due to incomplete data but were considered in sensitivity interpretation.

### 2.10 Ethical approval

This research was approved by the Ethics Committee of Jincheng People’s Hospital, Ethical Number: JCPH. No. 20250901002. The informed consent was waived because of the retrospective design. All the data were de-anonymized before analysis.

## 3 Results

### 3.1 Baseline characteristics according to Dietary Inflammatory Index

The final study comprised 500 patients with coronary heart disease (CHD) diagnosed by angiography. 61.8% of the group were men, and the study population was predominantly of Han Chinese ethnicity (>97%), and their average age was 60.9 ± 10.1 years. 38 months was the median follow-up period (interquartile range: 26–58 months). 63 fatalities (12.6%) were noted during follow-up, and 96 patients (19.2%) had at least one major adverse cardiovascular event (MACE).

Table 1 displays baseline clinical, biochemical, and demographic variables stratified by Dietary Inflammatory Index (DII) quartiles. Compared to individuals in the lowest DII quartile (Q1), those in the highest quartile (Q4) were considerably older and had a higher body mass index. Across DII quartiles, the prevalence of current smoking, diabetes mellitus, and hypertension increased gradually (all *p* for trend <0.01).

**Table 1:** Baseline characteristics of participants according to Dietary Inflammatory Index quartiles.

Variable	Q1 (*n* = 125)	Q2 (*n* = 125)	Q3 (*n* = 125)	Q4 (*n* = 125)	*p*-value
Age, years	57.9 ± 9.4	59.3 ± 9.7	61.4 ± 10.2	64.8 ± 10.5	<0.001
Male sex, %	60.0	61.6	62.4	63.2	0.52
Female sex, %	40.0	38.4	37.6	36.8	—
Race/ethnicity (Han Chinese, %)	98.4	97.6	98.4	97.6	0.88
BMI, kg/m^2^	26.7 ± 3.5	27.4 ± 3.7	28.0 ± 3.8	29.0 ± 4.1	<0.001
Diabetes mellitus, %	26.4	33.6	39.2	47.2	<0.001
Hypertension, %	51.2	57.6	64.0	71.2	<0.001
Dyslipidemia, %	54.4	61.6	69.6	76.0	<0.001
Total cholesterol, mg/dL	182 ± 36	191 ± 39	201 ± 42	213 ± 45	<0.001
LDL-C, mg/dL	104 ± 28	110 ± 30	118 ± 33	127 ± 36	<0.001
hs-CRP, mg/L	1.8 (1.1–2.7)	2.3 (1.4–3.4)	3.0 (1.9–4.3)	4.1 (2.8–5.9)	<0.001

Participants with greater pro-inflammatory food patterns had significantly higher levels of systemic inflammatory load, as shown by hs-CRP concentrations. On the other hand, statin and antiplatelet therapy were distributed similarly across quartiles.

It should be noted that participants with higher DII scores tended to be older, have higher BMI, and more prevalent comorbidities. Although these variables were adjusted for in multivariable models, baseline imbalances could contribute to residual confounding.

### 3.2 Dietary Inflammatory Index and individual components of MACE

Increasing DII quartiles were linked to increasingly greater occurrences of non-fatal myocardial infarction, ischemic stroke, and cardiovascular death when the individual components of MACE were examined (Table 2). Cardiovascular mortality showed the largest correlation, more than quadrupling from the lowest to the highest DII quartile (2.4% in Q1 vs. 11.2% in Q4; *p*-trend <0.001).

**Table 2:** Incidence of individual cardiovascular outcomes by DII quartiles.

Outcome	Q1 (%)	Q2 (%)	Q3 (%)	Q4 (%)	*p*-trend
Non-fatal MI	3.2	4.8	6.4	9.6	0.002
Ischemic stroke	2.4	3.2	5.6	8.0	0.001
CV death	2.4	4.0	6.4	11.2	<0.001
Composite MACE	11.2	15.2	20.8	29.6	<0.001

### 3.3 Dietary Inflammatory Index and major adverse cardiovascular events

**Figure 2:**
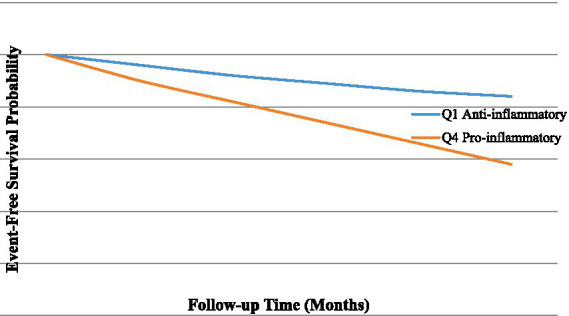
Kaplan–Meier event-free survival by Dietary Inflammatory Index.

As DII quartiles rose, the cumulative incidence of MACE gradually increased. In Q1, Q2, Q3, and Q4, the event rates were 11.2, 15.2, 20.8, and 29.6%, respectively (*p* for trend <0.001). Participants with stronger pro-inflammatory diets had significantly poorer event-free survival, according to Kaplan–Meier analysis (Figure 2).

Higher DII quartiles were linked to an elevated risk of MACE in Cox proportional hazards models (Table 3). Participants in Q4 exhibited an 82% greater risk of MACE than those in Q1 after full multivariable adjustment (HR 1.82, 95% CI 1.27–2.61), with a clear dose–response association.

**Table 3:** Association between Dietary Inflammatory Index quartiles and risk of MACE.

DII quartile	Unadjusted HR (95% CI)	Age- and sex-adjusted HR (95% CI)	Fully adjusted HR* (95% CI)
Q1 (ref)	1.00	1.00	1.00
Q2	1.31 (0.86–1.99)	1.27 (0.83–1.95)	1.22 (0.80–1.87)
Q3	1.88 (1.26–2.80)	1.79 (1.19–2.68)	1.64 (1.09–2.47)
Q4	2.71 (1.86–3.95)	2.34 (1.58–3.47)	1.82 (1.27–2.61)
*p*-trend	<0.001	<0.001	<0.001

*Adjusted for age, sex, BMI, smoking, diabetes, hypertension, dyslipidemia, medication use, and total energy intake.

### 3.4 Time-to-event analysis for MACE

Kaplan–Meier survival analysis demonstrated significantly lower event-free survival among participants with higher DII scores (log-rank *p* < 0.001). Divergence between survival curves became evident after approximately 18 months of follow-up and widened progressively thereafter. In multivariable Cox regression models (Table 4), each one-unit increase in DII score was associated with a 21% higher risk of MACE (HR 1.21, 95% CI 1.11–1.33).

**Table 4:** Cox proportional hazards models for risk of MACE per 1-unit increase in DII.

Model	HR per 1-unit increase in DII (95% CI)	*p*-value
Unadjusted	1.29 (1.18–1.41)	<0.001
Age- and sex-adjusted	1.24 (1.13–1.36)	<0.001
Fully adjusted*	1.21 (1.11–1.33)	<0.001

*Adjusted for age, sex, BMI, smoking status, diabetes, hypertension, dyslipidemia, medication use, and energy intake.

### 3.5 Dietary Inflammatory Index and all-cause mortality

All-cause mortality rose throughout the DII quartiles over the follow-up period. Q1, Q2, Q3, and Q4 mortality rates were 8.0, 10.4, 13.6, and 18.4%, respectively. Following complete adjustment, the risk of death was considerably higher for those in the highest DII quartile than for those in Q1 (HR 1.68, 95% CI 1.05–2.69) (Table 5).

**Table 5:** Dietary Inflammatory Index and risk of all-cause mortality.

DII quartile	Deaths, *n* (%)	Fully adjusted HR (95% CI)	*p*-value
Q1 (ref)	10 (8.0)	1.00	—
Q2	13 (10.4)	1.21 (0.66–2.22)	0.54
Q3	17 (13.6)	1.45 (0.83–2.55)	0.19
Q4	23 (18.4)	1.68 (1.05–2.69)	0.03
*p*-trend	—	—	0.02

### 3.6 Cardiac-related hospital readmissions

A total of 142 participants (28.4%) experienced at least one cardiac-related hospital readmission. A higher DII score was linked to a higher chance of readmission. After multivariable adjustment, the risks of readmission were almost twice as high for Q4 participants as for Q1 (OR 1.94, 95% CI 1.29–2.91) (Table 6).

**Table 6:** Adjusted odds ratios for cardiac-related readmissions.

DII quartile	Adjusted OR (95% CI)
Q1 (ref)	1.00
Q2	1.19 (0.77–1.84)
Q3	1.51 (1.00–2.29)
Q4	1.94 (1.29–2.91)
*p*-trend	<0.001

### 3.7 Association between Dietary Inflammatory Index and inflammatory biomarkers

Elevated hs-CRP concentrations were substantially correlated with higher DII values. There was a 0.31 mg/L rise in hs-CRP for every unit increase in DII (*β* = 0.31, *p* < 0.001), suggesting a possible inflammatory route connecting diet to worse cardiovascular outcomes. The DII score and hs-CRP values were found to be significantly positively correlated by linear regression analysis. All age and sex subgroups showed the same connection (interaction *p* > 0.10) (Table 7).

**Table 7:** Association between Dietary Inflammatory Index (DII) and circulating inflammatory biomarkers.

Biomarker	β per 1-unit DII (95% CI)	*p*-value
hs-CRP (mg/L)	0.31 (0.22–0.40)	<0.001
IL-6 (pg/mL)*	0.18 (0.09–0.27)	<0.001
TNF-α (pg/mL)*	0.11 (0.04–0.18)	0.002

The asterisk (*) indicates that IL-6 and TNF-α measurements were available only in a subset of participants and were analyzed accordingly.

### 3.8 Sensitivity and subgroup analyses

In sensitivity studies that excluded individuals with a history of myocardial infarction, heart failure, or chronic renal disease, the relationship between DII and MACE remained strong. Subgroup analyses showed consistent relationships between statin use, age (<65 vs. ≥65 years), sex, and BMI categories; no substantial impact modification was seen (Table 8).

**Table 8:** Subgroup analysis of the association between DII and risk of MACE.

Subgroup	HR per 1-unit DII (95% CI)	Interaction *p*
Male	1.20 (1.07–1.35)	0.62
Female	1.23 (1.08–1.40)	—
Age <65 years	1.19 (1.05–1.35)	0.58
Age ≥65 years	1.24 (1.10–1.41)	—
Statin users	1.18 (1.06–1.32)	0.44
Non-users	1.25 (1.10–1.43)	—

## 4 Discussion

This paper presents extensive evidence on the association between dietary inflammatory potential using the Dietary Inflammatory Index (DII) and poor clinical outcomes in 500 patients with angiographically verified coronary heart disease (CHD). We have shown that high DII scores are highly related to the risk of major adverse cardiovascular events (MACE), all-cause mortality, cardiac-related hospital readmission, and high levels of systemic inflammation, which underscores the importance of pro-inflammatory nutritional patterns in the pathophysiology of CHD.

We found that the participants in increased DII quartiles were older and had higher BMI, and more frequent comorbidities, such as diabetes mellitus, hypertension, and dyslipidemia. This risk factor malalignment is consistent with previous investigations (14, 15) and implies that pro-inflammatory diets can increase the effect of conventional heart risk factors. In addition, increased DII scores were always linked to increased inflammatory biomarkers, such as hs-CRP, IL-6, and TNF-a. High levels of hs-CRP were in specific, and the levels increased by 0.31 mg/L with each 1-unit change in DII. The findings support mechanistic theories of mechanisms that support chronic low-grade inflammation, endothelial dysfunction, oxidative stress, and atherogenesis by pro-inflammatory diets, which are likewise supported by other mechanistic studies (16–18).

High DII scores were associated with higher rates of non-fatal myocardial infarction, ischemic stroke, cardiovascular death, and composite MACE. It is worth noting that the dose–response relationship was strong as cardiovascular mortality more than four-fold between Q1 and Q4. These are in line with the findings by Zhao et al. (17) and Bodén et al. (19), who have indicated that pro-inflammatory diets cause plaque vulnerability, thrombotic risk, and cardiovascular adverse events. Our findings demonstrate that dietary inflammation may be associated with cardiovascular outcomes that are independent of conventional risk factors because DII may serve as a marker associated with cardiovascular outcomes even after adjustments for confounders (age, sex, BMI, comorbidities, medication use, and energy intake). The Kaplan–Meier analyses of survival showed that the event-free survival of the participants whose DII scores were higher increased gradually and substantial divergence among the curves of the survival is observed after 18 months. Cox proportional hazards models adjusted fully showed that Q4 participants were at a higher risk of MACE 82 per cent (HR 1.82, 95% CI 1.27–2.61). The relationship between DII and the increased risk of MACE was found to be continuous, with a risk that increased 21% with every 1-unit of DII. The findings are consistent with other cohort studies done in the past by Chen et al. (20), Gao et al. (21), and Wang et al. (22) and support the clinical applicability of DII as a quantitative cardiovascular-related risk factor.

There was an all-cause mortality increase in all DII quartiles with Q4 participants having 18.4% versus 8.0% mortality in Q1. Also, the increased DII scores were linked to a little less than twice higher risk of hospital readmissions of cardiac nature (Q4 OR 1.94, 95% CI 1.29–2.91). These data reflect the previous results of Li et al. (23) and Wang et al. (22) that instead of being a cause of acute events, pro-inflammatory diets may be associated with worse prognosis and healthcare use among CHD patients in the long run. This highlights the possible utility of dietary interventions as a cost-saving option of secondary prevention. Our research offers to a large extent of mechanistic data about the relationship between dietary inflammation and systemic inflammatory activation. DII has a positive correlation with hs-CRP, IL-6 and TNF-a indicating that pro-inflammatory diets could enhance vascular inflammation, endothelial dysfunction and oxidative stress, which play key parts in plaque formation and destabilization. These results agree with the mechanistic mechanisms that have been theorized in Ramezankhani et al. (16) and Ni et al. (18) and suggest that dietary inflammation may play a direct role in atherosclerotic process. In addition, the fact that these associations are similar in age, sex, and subgroups in terms of statin-use indicates that the effect of dietary inflammation is widespread and is not conditioned by other modulating factors.

The strength of the DII-MACE association was ensured by sensitivity analyses that eliminated those who had suffered a heart attack or heart failure or had a history of chronic kidney disease. The results of the subgroup analyses that were stratified by age, sex, BMI, and statin use showed that there were consistent relationships, and no significant effect modification. This helps to generalize the findings of our research and is consistent with the results of Pan et al. (24) and Tyrovolas et al. (25), who indicate that dietary inflammatory potential is a valid predictor of cardiovascular risk in a range of patient groups (24, 25). Our results are generally in line with other cohort studies that have been done associating DII to cardiovascular outcomes. Nevertheless, this research builds on previous literature by targeting the patients with angiography-proven CHD, longitudinal follow-up, and clinical and biomarker outcomes. This is how this holistic methodology supports causal inference and clinical interest of the relationship between dietary inflammation and CHD outcomes (26–28).

Our findings highlight the potential usefulness of DII as a marker to identify individuals at higher risk of adverse outcomes in CHD patients. Dietary inflammatory potential could be reduced by the use of nutritional counseling that focuses on anti-inflammatory foods and eliminates pro-inflammatory foods to lower MACE, mortality, and use of health products. It might be the most useful strategy when dealing with high-risk populations with high systemic inflammation or comorbid metabolic conditions. Moreover, the results justify the introduction of DII into the regular clinical evaluation and emphasize the necessity of the need-specific nutrition intervention in combination with drug treatment. Randomized controlled trials should be conducted in future to establish the possibility of reducing inflammatory potentials of diet which can be translated into quantifiable cardiovascular events and mortality. These results justify the incorporation of dietary inflammatory measurement within the usual cardiovascular risk stratification and indicate the promise of the use of anti-inflammatory dietary approaches as adjunctive prevention in the secondary prevention.

### 4.1 Strengths of the study

There are a number of strengths in this study. First, the study population of 500 patients with angiographically proven coronary heart disease is well characterized, which increases accuracy in diagnosis and reduces the possibility of outcome misclassification. Second, based on the application of Dietary Inflammatory Index (DII), a validated, standardized scale of inflammatory potential of the dietary intake, it is possible to meaningfully compare the result with the existing international literature. Third, major adverse cardiovascular events (MACE), all-cause mortality, inflammatory biomarkers, and cardiac-related hospital readmission were included in the study as a set of outcomes, which allowed evaluating both clinical and biologic outcomes in the holistic way. Fourth, the presence of a rather long median follow-up time (38 months) enhanced the time-to-event results and made it possible to make strong Kaplan–Meier and Cox proportional hazards analyses. Lastly, the high quality of the findings was improved by a comprehensive multivariate adjustment of the demographic, clinical, biochemical, and treatment-associated confounders, sensitivity and subgroup analyses.

### 4.2 Limitations of the study

Various limitations are associated with this research. To start with, the retrospective observational design is not capable of causal inference, and conclusions are to be made carefully. Second, even after multivariate adjustment, it is possible that there is residual confounding especially by factors that are not measured (socioeconomic status, physical activity, general diet quality, and medication adherence) and incomplete adjustment for socioeconomic variables such as income and education may have further contributed to this limitation. Third, dietary intake was measured baseline only by the use of an FFQ, which is subject to recall bias and not time-varying. Fourth, the DII has been obtained based on fewer components, which can imply an impact on accuracy. Fifth, the study was a single-centered one and had a rather homogeneous population, which might not be generalizable. Besides, the outcome assessment was dependent on hospital records, which might not be able to include out-of-hospital cardiovascular events, which could underestimate the rate of events. Also, insufficient information regarding the attendance of cardiac rehabilitation programs did not allow the research to adjust the potentially significant factor impacting the cardiovascular outcomes. Finally, there were no other inflammatory biomarkers besides hs-CRP that could have further mechanistically interpreted the results.

### 4.3 Future recommendations

Future studies ought to be done on prospective, multicenter cohort studies and randomized controlled trials to prove causality of inflammatory potential of diet and cardiovascular outcomes. Frequent longitudinal dietary evaluations would enable the measurement of the changes in DII and their effect on risk courses. To establish whether a reduction in DII is associated with significant decreases in MACE, mortality, and hospital readmissions, interventional studies that aim at reduction of dietary inflammatory load (e.g., adherence to Mediterranean or plant-based dietary patterns) are necessary. Also, the subsequent research should investigate interactions between genes and dietary practices and microbiomes and dietary practices, which can adjust the inflammatory reactions to dietary habits. The inclusion of DII into models and clinical decision making tools of cardiovascular risk prediction can also be used to personalize diets interventions in partnership with secondary prevention.

## 5 Conclusion

This paper shows that there is a positive association between the Dietary Inflammatory Index and the risk of major adverse cardiovascular events, all-cause mortality, cardiac hospital readmission, and systemic inflammation in coronary heart disease patients. The patterns of dose–response and correlation with inflammatory biomarkers indicate that there may be a causal relationship between pro-inflammatory diets and negative cardiovascular effects, but this cannot be causally attributed. These results highlight dietary inflammation as a potentially modifiable factor in CHD, warranting further investigation and supporting dietary interventions to reduce inflammatory burden and improve long-term prognosis.

## Data Availability

The raw data supporting the conclusions of this article will be made available by the authors, without undue reservation.
